# P01.24. Central processing by electroacupuncture of vasodepression and bradycardia reflex responses

**DOI:** 10.1186/1472-6882-12-S1-P24

**Published:** 2012-06-12

**Authors:** S Tjen-A-Looi, P Li, J Longhurst

**Affiliations:** 1 University of California, Irvine, Irvine, USA

## Purpose

Electroacupuncture (EA) at P5-6 reduces sympathoexcitatory blood pressure (BP) reflex responses. Gamma-aminobutyric acid (GABA) receptors in the brainstem region rostral ventral lateral medulla (rVLM) contribute to modulation of sympathoexcitatory visceral reflexes during EA. Gastric distension in hypercapnic acidotic rats, by activating both sympathetic and vagal afferents, decreases BP and heart rate (HR) through a GABA_A_ mechanism in the rVLM. This study investigated the hypothesis that EA modulates gastric induced hemodynamic depressor responses through actions in nuclei that process both sympathetic and parasympathetic outflow.

## Methods

Anesthetized and hypercapnic acidotic induced rats were used to examine the central processing of the actions of EA. An unstressed 2-cm diameter latex balloon attached to a polyurethane tube was inserted into the stomach through the mouth and esophagus to induce gastric distention. Acupuncture needles were placed near the wrist at P5-6 acupoints for 30-min EA (2 Hz, 0.2-0.4 mA, 0.5 ms).

## Results

We observed repeatable decreases in BP and HR with gastric distention every 10 min. Bilateral EA at P5-6 for 30 min reversed the hypotensive response from -26±3 to -6±1 mmHg and the bradycardia from -35±11 to -10±3 beats/min for over 70 min. EA’s action on decreased BP and HR, respectively, was inhibited by microinjection of gabazine, a GABA_A_ receptor antagonist, into the caudal–VLM (cVLM) or the nucleus ambiguus (NAmb). Gabazine microinjected into the rVLM reversed EA action on both depressor and bradycardia responses.

## Conclusion

Thus, EA through GABA_A_ receptor mechanisms modulates reflex sympathoinhibition and vagal excitation leading to cardiovascular depression through actions in the rVLM, cVLM and NAmb. These data indicate that EA can normalize elevated and depressed blood pressure.

